# Mental Health during the Second Wave of the COVID-19 Pandemic—Polish Studies

**DOI:** 10.3390/ijerph18073423

**Published:** 2021-03-25

**Authors:** Jan Chodkiewicz, Joanna Miniszewska, Emilia Krajewska, Przemysław Biliński

**Affiliations:** 1 Institute of Psychology, University of Lodz, 91-433 Lodz, Poland; joanna.miniszewska@now.uni.lodz.pl (J.M.); emilia.krajewska@edu.uni.lodz.pl (E.K.); 2 The President Stanisław Wojciechowski State University of Applied Sciences in Kalisz, 62-800 Kalisz, Poland; bildom@gmail.com; 3 Copernicus Memorial Multidisciplinary Comprehensive Cancer and Traumatology Center Lodz, 93-513 Lodz, Poland

**Keywords:** mental health, stress, COVID-19 second wave

## Abstract

The presented research aimed to identify the impacts of the second wave of the coronavirus disease 2019 (COVID-19) pandemic on respondents’ mental health state and identify variables related to the respondents’ symptoms of anxiety and depression; 618 subjects participated in the research. A specially prepared survey and Polish adaptations of the following methods were used: Hospital Anxiety and Depression Scale (HADS), Perceived Stress Scale (PSS 10), MINI-COPE Questionnaire (Brief COPE Inventory), Alcohol Use Disorder Identification Test (AUDIT), Scale of Death Anxiety (SDA), The Fear of COVID-19 Scale (FCV-19S). Over 24% of the respondents reported having experienced suicidal thoughts since the start of the pandemic. Almost 16% drank alcohol in a risky or harmful way. The average value of perceived stress indicated its high and very high intensity. Over 20% had symptoms of anxiety disorders, and almost 19% had anxiety and depression symptoms. It means that almost 40% of the respondents probably have mental disorders. More women, younger people, and those with disorders prior to the onset of the pandemic were among those who manifested these disorders. They also used passive and avoidance stress coping strategies more frequently. In conclusion, the second wave of the pandemic negatively affected the mental health of the respondents. A high percentage of the respondents manifested anxiety and anxiety-depressive disorders and declared having of suicidal thoughts.

## 1. Introduction

The coronavirus disease 2019 (COVID-19) pandemic has already led to and will still, inevitably, lead to numerous negative consequences: economic, social, connected with health policy and associated with physical and mental health. Reports from the beginning of the pandemic and earlier ones, concerning previous epidemics (e.g., Ebola 2014/2016, H1N1influenza 2009/2010, bird flu 2006, severe acute respiratory syndrome -SARS 2003), indicate that the experience of large scale catastrophes is accompanied by increases in depression and anxiety, posttraumatic stress disorder (PTSD) and a broad spectrum of other mental disorders, including addictions [1,2,3,4,5,6,7,8,9,10,11]. Salari et al. [12], on the basis of a systematic review and meta-analysis of reports on anxiety and depression during the first wave of the COVID-19 pandemic confirmed the prevalence of the aforementioned disorders and pointed towards a significant increase in suicidal thoughts and panic attacks among their symptoms. The outcomes manifested as mental health changes, are already being referred to as “the inevitable next pandemic” or “the pandemic of severe mental disorders” [3,13,14,15]. Therefore, it is of great importance to address long- and short-term aftermaths of the pandemic, both on the individual and population level, which is also underlined by the United Nations (UN), among others [16]. However, we are still unaware of the extent of the pandemic’s impact on mental health and we do not know the dynamics of these changes, whether they intensify or weaken as we adapt to specific pandemic-related restrictions. We also lack data concerning psychological resources (adaptive stress coping strategies) which may be of key importance in strengthening and improving mental health. In other words, it seems vital to find out and identify the properties which will help us better understand psychological mechanisms of anxiety and depression in the era of the COVID-19 pandemic, and enable us to outline guidelines for therapeutic work. During the first wave of the pandemic, we carried our research into mental health and the study revealed that approximately 10% of respondents suffered from suicidal thoughts occurring since the lockdown began, and over 25% reported significant mental health deterioration [17]. Therefore, it seems interesting to analyse mental functioning of the respondents during the second wave of the coronavirus pandemic, which in Poland started in October 2020, when a sudden increase in coronavirus cases and COVID-related deaths were reported and the restrictions, partially lifted for summer months, were imposed again. The scale of changes is illustrated by the following data: during the first coronavirus wave in April 2020, there were on average 324 COVID cases per day, in September the daily case number was 805, and in October over 8500. In November, the seven-day average number of new cases exceeded 20,000 [18].

The research aimed to:

(a) identify the impacts of the second wave on respondents’ mental health state during the second wave of the COVID pandemic in Poland (November–beginning of December 2020);
(b) identify of variables related to the respondents’ symptoms of anxiety and depression.

## 2. Sample

The study was carried out between 10 November and 5 December 2020.At that time the levels of new coronavirus cases and COVID-related deaths were very high, and the imposed restrictions really strict (closure of shops, restaurants, pubs, no social meetings). Due to the epidemiological situation, the research was conducted online on adult participants recruited by the “snowball” method (the participants sent each other a link to the survey) [19]. It was conducted fully anonymously by means of a Google Form and shared by the researchers on Facebook. Information about the research procedure and links can be found on the State Agency for the Prevention of Alcohol-Related Problems in Poland (PARPA) website. Each of the participants could opt out of the research at any time and had the opportunity to see the results of the tests performed. The research was approved by the Bioethics Scientific Research Committee of the University of Lodz.

The research procedure was conducted in accordance with the Declaration of Helsinki of the World Medical Association [20] and the ethical codes of the Belmont Report [21]. The study was accepted by the State Agency for the Prevention of Alcohol-Related Problems (PARPA) in Poland.

There were 618 respondents who participated in the research: 500 women (80.9%) and 118 men (19.1%). The respondents’ mean overall age was 26.04 (standard deviation (SD) = 9.74). The youngest participant was 18, the oldest one 76 years old.

Table 1 shows that the study participants were mostly large city dwellers, single, with higher education, no children, learning or studying.

## 3. Descriptive Statistics of the Sample

The respondents were asked several questions concerning their functioning during the pandemic, the use of psychoactive substances, and mental health. The most important information is presented in Table 2, Table 3 and Table 4.

Table 2 shows that most participants stayed at home, went our occasionally, were neither in quarantine nor tested positive for COVID-19.

At the same time, over 40% declared that someone from their family and friends had received a positive test result. Moreover, 28 people (4.53%) faced the death of loved ones due to COVID-19.

Table 3 shows that the psychoactive substance most often used by the respondents was alcohol (consumed by almost 75% of respondents), then tobacco (smoked by over 30%). Only few respondents (almost 7%) admitted having taken drugs. At the same time, almost 30% claimed that the pandemic had changed their drinking habits: some drank less, while others more. 15% of tobacco smokers signalled a change in their addiction (they smoked less/more). It is noteworthy that the percentage of people reporting the occurrence of suicidal thoughts since the pandemic began (24%) is highly alarming. The positive finding was that over 90% of respondents did not take any drugs at all.

Table 4 presents the most important data concerning the participants’ somatic and psychiatric health. Most respondents did not report any serious somatic or psychiatric diseases and did not attempt to commit suicide in the past. What is both interesting and causes concern is the result showing a high rate of addiction to alcohol in the participants’ families. It is noteworthy that almost every third respondent reported current or past psychiatric disorders.

## 4. Methods

In the research we used:

1. Hospital Anxiety and Depression Scale (HADS) designed by Zigmond and Snaith [22,23] and commonly used in screening for psychiatric disorders. The method contains two scales to assess anxiety and depression, each one includes seven statements. The questions refer to the subject’s well-being during the previous week. Two additional questions about the level of anger were added to the questionnaire. Responses were rated on a four-point Likert scale (0–3), so the final result for each subscale ranged from 0–21 points. The result in case of the two questions concerning aggression ranged between 0–6 points. According to the Polish norms, a score between 0–7 points means no disorders, between 8–10 points represents a borderline, whereas a score between 11–21 points indicates a disorder [23]. Cronbach’s α in this research totalled 0.84 for anxiety and 0.83 for depression.
2. Perceived Stress Scale (PSS 10) designed by Cohen, Kamarck and Mermelstein [24]. The questionnaire comprises 10 questions for assessing the intensity of stress related to participants’ life situation during the last month. Respondents use a 5-point frequency scale (0–4) with categories of answers ranging from “never” to “very often”. In our research sample, the method proved to be satisfactorily reliable: Cronbach’s α of 0.84. This method has its Polish adaptation and Polish sten norms with scores over 20 points (sten 7 and above) considered to be high [25].
3. MINI-COPE Questionnaire (Brief COPE Inventory) designed by Carver [26]. It includes 28 statements which compose 14 coping strategies (two statements in each strategy): active coping, planning, positive reframing, acceptance, humour, religion, use of emotional support, use of instrumental support, self-distraction, denial, venting, substance use, behavioural disengagement, self-blame. Our respondents selected one out of four possible replies ranging in scores from “I almost never do this” (0 points) to “I almost always do this” (3 points). Each of the 14 coping strategies is assessed separately and the higher the score, the more often it is used. This method is used to assess dispositional or situational coping, and the latter option was used in the presented analyses (coping in a pandemic situation). This method has its Polish version and its internal compliance is 0.86 [27].
4. Alcohol Use Disorder Identification Test (AUDIT), developed by the World Health Organization [28] and used to diagnose alcohol consumption habits. This test was devised as a screening tool identifying people whose alcohol consumption has become risky, harmful or indicative of addiction. The construction of this method was based on research carried out in numerous countries which demonstrated that certain symptoms (e.g., regular consumption of large amounts of alcohol, alcohol-related violation of social norms, alcohol-related injuries) may be early signs of alcohol problems and a developing addiction. As a result of analyses, and aspiring to isolate symptoms that would be common to different countries and cultures, the researchers included 10 questions in the test: three on the quantity and frequency of drinking (1–3), three on the addiction to alcohol (4–6), and four on the problems causes by alcohol (7–10). Total scores of 8–15 points indicate likely hazardous drinking, 16–19 points harmful drinking, 20 points and more a likely addiction to alcohol. This was checked by comparing AUDIT results across many countries and cultures, and with external measures (including opinions of specialists) [28].
5. Scale of Death Anxiety (SDA), designed by Cai et al. [29]. This method consists of 17 items and assesses the intensity of death anxiety (thoughts, emotions, avoidance) present within the last month. Cronbach’s α in this research totalled 0.94 for the whole scale. The method was adapted to Polish conditions [30].
6. The Fear of COVID-19 Scale (FCV-19S), designed by Ahorsu et al. [31]. It consists of items obtained from a comprehensive review of existing scales of anxiety, expert ratings, and interviews with research subjects. As a result of the applied statistical analysis, 7 items were selected. All of them correlated with the overall result in a range of 0.47 to 0.56 and characterized by factor load from 0.66 to 0.74. All items have a 5-point Likert scale ranging from: “I disagree” (1)–(5) “I strongly agree”, and results may range from 7 to 35 points. Cronbach’s alpha of the original version was 0. 82. In this research it was 0.93. The method was adapted to Polish conditions [30].

We also used a survey with several questions on the use of psychoactive substances, mental and somatic health, wellbeing, and behaviours during the pandemic.

T-test analysis of variance (ANOVA) and chi-square test were used for the statistical analyses. Calculations were carried out using the STATISTICA (Statistica 13, StatSoft Poland) software.

## 5. Results

The first question concerned participants’ mental health during the second wave of the pandemic. Table 5 presents mean scores in HADS, PSS10 and AUDIT scales.

The results obtained by means of methods analysing the state of mental health during the previous week (HADS) and the level of stress within the last month (PSS10) were compared with the Polish norms [23,26]. The anxiety level in HADS, M = 10.05, is a borderline, which proves an increased level of stress in the study group. A thorough analysis shows that the scores of 202 respondents (32.69%) meant anxiety disorders (>11 points). The mean score of depression M = 6.98 is within the broad norm. However, the scores of 123 respondents (23.14%) were indicative of depressive disorders. The mean results obtained by means of the Perceived Stress Scale are within the range of high scores (8 sten), which means a high level of perceived stress during the last month. The mean result obtained in AUDIT is within the norm, and detailed analysis shows that 96 respondents (15.86%) consumed alcohol in a hazardous or harmful manner (result > 8 points).

To analyse the correlation between the state of mental health and death anxiety, fear of coronavirus, the level of perceived stress, alcohol consumption and pandemic stress coping strategies in the study period, the respondents were divided into 4 groups–respondents with anxiety and depression scores within the norm (group 1, *n* = 344–55.66%), whose results indicate anxiety disorders only (group 2, *n* = 131–21.20%), depression (group 3, *n* = 26–4.21%), and those whose scores show a simultaneous occurrence of anxiety and depressive disorders (group 4, *n* = 117–18.93%).

Table 6 presents the comparison of these groups using the ANOVA test (post hoc Tukey RIR test for uneven n’s).

As Table 6 shows, there are a number of statistically significant differences between the study groups.

Respondents with both anxiety and depressive disorders (group 4) are in the worst situation. They are younger and much more scared of coronavirus than respondents without disorders (group 1). They also declare greater death anxiety compared to respondents with no disorders and with depressive disorders (groups 1 and 2), and the highest level of stress. Regarding pandemic stress coping strategies, they are considerably less likely than respondents from groups 1 and 2 to use positive reframing, acceptance and self-distracting. However, they more often blame themselves for the situation and discontinue any actions. They are also less likely to cope in an active way or seek instrumental or emotional support compared to respondents from group 1 (with no disorders). On the other hand, they vent emotions more often.

Respondents with anxiety, compared to group 1 (with no disorders), are younger and signal a higher level of death anxiety, stress, and fear of coronavirus. They more often vent emotions, deny the situation, and disengage. Compared to respondents with the symptoms of both anxiety and depression (group 4), the latter ones more often use positive reframing and acceptance of the difficult situation, but also self-blame. Compared to group 3, respondents declaring symptoms of depression, those with anxiety are more likely to seek emotional and instrumental support.

Depressive respondents exhibit higher death anxiety and stress than those who do not declare disorders (group 1). Moreover, they are considerably less likely to seek emotional and instrumental support (also when compared to respondents with anxiety). On the other hand, they more often use self-blame and behavioural disengagement.

It is also worth noting the characteristics of the first groups, i.e., those respondents who do not declare any symptoms of anxiety or/and depressive disorders. They are older, with a lower level of fear of coronavirus (who are different from respondents with anxiety and with disorders of both categories in a statistically significant way). They are considerably less likely to show death anxiety and their stress level is lower than that of the remaining groups. They also differ in that they do not stop acting when faced with stress and are less prone to self-blame for their failures.

We also analysed relationships between sociodemographic variables and belonging to groups of diverse occurrence of mental disorders (chi-square test with Yates’ correction). The aforementioned analysis showed that gender and education influence the belonging to these groups in a significant way (*p* > 0.01); disorders are more common in women compared to men, and in respondents with secondary level education compared to those with higher education. No differences were observed regarding place of residence (*p* = 0.18), marital status (*p* = 0.11), having children (*p* = 0.22), and employment (*p* = 0.14).

Considering variables connected with the current situation, the groups differed only concerning the experience of quarantine (*p* = 0.04)–those who had been in quarantine functioned worse than those who had not. No differences were observed regarding current living conditions (*p* = 0.33) and, interestingly, with regard to being tested positive for coronavirus (*p* = 0.61), having a family member tested positive (*p* = 0.66), and in case of the death of loved ones (*p* = 0.61).

Just as we expected, very significant differences were observed between groups when mental health was taken into consideration. Anxiety and depressive disorders were connected with experiencing suicidal thoughts (*p* < 0.001), consuming larger amounts of alcohol (*p* < 0.01), and more frequent smoking (*p* < 0.01). No correlations were observed regarding drug use (*p* = 0.11), but the number of respondents who admitted taking drugs was very low. The analysed disorders show correlations with suicidal attempts in the past (*p* > 0.001) as well as with current or previous psychiatric disorders (*p* > 0.001). No differences were observed regarding somatic diseases (*p* = 0.33) or parents’ addictions (*p* = 0.22).

## 6. Discussion

This study indicates that COVID-19 pandemic has serious consequences for mental health, which are manifested as intensified psychopathological symptoms in many people. In the studied group from Poland, 24% declared the occurrence of suicidal thoughts since the beginning of the pandemic. It is worth paying attention to the research conducted in the USA in June 2020, in which the percentage of respondents seriously considering suicide in the last 30 days was 10.7%, but in the case of young people (18–24) it was 25.5% [32]. Moreover, research shows that 21% experienced symptoms indicative of anxiety disorders, and more than 4% of depressive disorders only. It is worrying that another 19% of respondents received results indicative of both anxiety and depressive disorders. This is supported by the comparison of the obtained results with the standards for HADS [23]. Thus, a total of 44% of the respondents manifested various disorders of mental functioning. The subject literature provides also information about different proportions concerning the distribution of the analysed disorders, depending on the country or even the province where the study was conducted—ranging from 6.33% to 50.9% in the case of anxiety disorders and from 14.6% to 48.3% in the case of depression [33].

It is important to note, however, that the state of people’s mental health deteriorated as the pandemic continues, which is demonstrated in a few reports [15,34,35]. However, it needs to be emphasized that different research tools were used with regard to psychiatric disorders. In our previous studies [17], the level of mean stress intensity was 18.96; SD = 6.63 (compared to M = 20.65; SD = 6.20 in this study), and the results denoting psychiatric disorders in 26.18% of respondents (compared to 44% in this study); 10% of respondents reported suicidal thoughts in the previous studies compared to as many as 24% in this one. Thus, we can also conclude that the mental functioning of the respondents deteriorated. However, it should ultimately be confirmed in longitudinal studies. Of course, reports from longitudinal studies have not been published yet and we can only speculate. However, it should be noted that our studies deal with the second wave of the pandemic and, therefore, the results can be compared to previous reports about the first wave. 

Our research also indicates a high level of perceived stress, which is in line with other reports e.g., [5], and signals the need to monitor a potential occurrence of psychosomatic disorders in successive studies. Carefully analysing our results with reference to Selye’s concept of stress [36], we suspect that, after initial mobilization and the subsequent stage of resistance, its level will decrease and it will be expressed through psychosomatic disorders.

The results we have obtained show that respondents with both anxiety and depressive disorders are in the worst situation. They feel the strongest death anxiety and fear of coronavirus. Importantly, they are also younger. Previous reports also indicated that younger adults were under greater mental strain during the pandemic. Research conducted on a representative sample of university students in China [37] demonstrated that their level of anxiety was higher compared to the general population. Odriozola-Gonzáleza et al. [38] observed a higher level of depression, anxiety, and stress in students compared to university staff. Kar, Kar and Kar [5] showed that the highest rate of mental health problems appeared in 20–30 year-olds, single, with higher education. Other researches [12,39,40,41,42,43,44] also came to this conclusion which is interesting because younger people do not belong to the group at risk of dying from COVID-19. It is suspected that their mental state results from their fear for the future and for their career, or it is connected with their inexperience in coping with difficult or unpredictable situations. It is also suggested that younger people more often use social media and share more negative information [12]. Perhaps young people are also worried about their own and their loved ones lives.

Another conclusion concerns a much less often use of positive reframing as a strategy for coping with the pandemic situation in respondents with mixed disorders. The analysed people without any disorders turned to that strategy much more often. In their studies, Çetin, Dönmez i Türkkan [45], also emphasise positive reframing in coping with the pandemic stress, presenting evidence that it is connected with a lower level of perceived stress. Other authors e.g., [45] also suggest the learning of positive reevaluation as a way to reduce the stress associated with experiencing a pandemic. It seems that another important and positive strategy used in coping with the pandemic stress is seeking social support—both emotional and instrumental. This conclusion can be drawn on the basis of research conducted in the Russian Federation by Medvedeva, Enikolopov, Boyko, Vorontsova [35].

This study also showed that anxiety and depressive disorders are associated with having suicidal thoughts, drinking larger amounts of alcohol and more frequent smoking. These relations, however, seem obvious and clear. Moreover, the analysed disorders show relationships with the occurrence of suicidal behaviours in the past and psychiatric disorders signalled currently or in the past. A similar regularity was observed by Kar, Kar and Kar [5].

In our study group, women coped with the pandemic situation with regard to mental health in a considerably worse way. This regularity was also confirmed in research conducted in groups of other nationalities [5,34,37,41,44,45]. Also the result obtained by us, indicating worse functioning of people who have undergone quarantine compared to people who did not have such experiences, was already signalled in the literature on the subject [46].

Another regularity is connected with respondents’ education; this research showed that, with regard to mental health, participants with secondary education coped with the pandemic situation in the worst way. However, other reports demonstrated that the problem is more common among people with a higher level of education, which means greater awareness and knowledge about coronavirus [12,37,41].

This research has certain limitations; first of all, it is not longitudinal. We do not have a baseline (pre-pandemic) measure so our assumptions are limited. We also do not know the prevalence of suicidal thoughts in the study group before the pandemic period. Longitudinal research could be beneficial in this regard [47]. Although it concerns the second wave of the pandemic, it does not show the dynamics of changes and only presents the respondents’ mental state estimated in a given period of time. Moreover, the research was conducted by means of the Internet and, therefore, our sample consisted only of people who were interested in giving responses. The group was also relatively small with an underrepresentation of men and people with primary/middle school and vocational education. As a result, the sample was not representative and we cannot generalise the results.

## 7. Conclusions

Research shows that the second wave of the pandemic negatively affected the mental health of the respondents. A high percentage of the respondents manifested anxiety and anxiety-depressive disorders and declared having suicidal thoughts.

The analysed disorders show relationships between gender, education, increased consumption of alcohol, smoking, and the range of strategies for coping with the pandemic stress.

The results we obtained indicate that it is justified to identify people with the symptoms of anxiety and depression (particularly occurring simultaneously) to provide them with additional psychological and therapeutic support in order not to further exacerbate the disorder but to reduce and eliminate it.

## Figures and Tables

**Table 1:** Characteristics of the sample: sociodemographic variables.

Sociodemographic Variables	*n* = 618	%
Place of residence		
Rural areas	152	24.60
Town < 100,000 inhabitants	309	25.40
Cities > 100,000 inhabitants	618	50.00
Education		
Primary/middle school	47	7.61
Vocational	11	1.78
Secondary level	306	49.51
Higher	254	41.10
Marital status		
Single	402	65.04
Married/partner	193	31.22
Divorced/separated	17	2.75
Widowed	6	0.97
Children		
Yes	104	16.83
No	514	83.17
Employment		
Studying/learning	238	38.51
Studying and working	123	19.90
Working full-time	184	29.77
Working occasionally	29	4.69
Unemployed	44	7.12

**Table 2:** Daily functioning during the pandemic.

Daily Functioning	*n* = 618	%
Current residence/work conditions		
I stay at home and I do not go outside at all	50	8.09
I stay at home and go out occasionally (walk, shopping)	333	53.88
I stay at home, but I go to work regularly	211	34.14
I go outside (friends, family)	24	3.88
When filling in the test, are you or have you been in quarantine for two weeks?		
Yes	118	19.09
No	500	80.91
Are you currently or were you tested positive for COVID-19?		
Yes	52	8.41
No	566	91.59
Is or was anyone from your family and friends tested positive for COVID-19		
Yes	267	43.20
No	351	56.80

**Table 3:** Mental functioning during the epidemic.

Mental Functioning	*n* = 618	%
Have you observed any suicidal thoughts in you since the introduction of the pandemic-related restrictions?		
Yes	146	23.62
No	472	76.38
If you drink, has your alcohol consumption changed since the introduction of the pandemic-related restrictions?		
No, it is the same	286	46.28
Yes, I drink less	113	18.28
Yes, I drink more	61	9.87
I do not drink alcohol	158	25.57
If you smoke, has your tobacco consumption changed since the introduction of the pandemic-related restrictions?		
No, it is the same	104	16.83
Yes, I smoke less	36	5.83
Yes, I smoke more	59	9.55
I don’t smoke	419	67.80
If you take drugs, has your drug use changed since the introduction of the pandemic-related restrictions?		
No, it is the same	23	3.72
Yes, I take more drugs	4	0.65
Yes, I take fewer drugs	16	2.59
I don’t take drugs	575	93.04

**Table 4:** Somatic and mental health of the subjects.

Somatic and Mental Health	*n* = 618	%
Are you or have you been suffering within the last year from any serious somatic diseases (e.g., diabetes, hypertension, malignancies)?		
Yes	55	8.90
No	563	91.10
Are you or have you been suffering from any mental disorders (e.g., depression, neurosis, eating disorder)?		
Yes	176	28.48
No	442	71.52
Have you ever attempted to commit suicide?		
Yes	73	11.81
No	545	88.19
Has any of your close relatives been addicted to alcohol?		
Yes	285	46.12
No	333	53.88

**Table 5:** The state of mental health and the level of perceived stress and AUDIT in the study group.

Variables	M	SD	Min.	Max.
HADS ANXIETY	10.05	5.28	0	21
HADS DEPRESSION	6.98	4.55	0	21
HADS ANGER	3.40	1.75	0	6
The Perceived Stress Scale (PSS-10)	24.67	7.49	5	40
AUDIT	4.25	3.89	0	23
Fear of coronavirus	15.81	5.73	7	35
Death anxiety	35.45	14.42	17	83

HADS—Hospital Anxiety and Depression Scale; PSS-10—The Perceived Stress Scale; AUDIT—Alcohol Use Disorder Identification Test; Fear of coronavirus—FCV-19S; Death Anxiety—SDA; M—mean; SD—standard deviation; Min.—minimum value; Max.—maximum value.

**Table 6:** Comparison of groups.

Variables	Group 1 *n* = 344		Group 2 *n* = 131		Group 3 *n* = 26		Group 4 *n* = 117		F	Post-Hoc Test
	M	SD	M	SD	M	SD	M	SD		
Age	27.46	10,35	23.28	7.41	26.58	12.09	24.50	8.80	7.15 **	1 > 2,4
Fear of coronavirus	14.85	5.14	17.39	6.07	14.80	6.00	17.09	6.28	9.13 **	1 < 2,4
Death anxiety	29.39	10.93	42.85	14.99	37.30	14.72	44.54	14.05	61.29 **	1 < 2,3,4
										3 < 4 ^1^
AUDIT	4.07	3.60	4.00	3.44	4.96	4.77	4.91	4.75	1.81	
PSS 10	20.65	6.20	28.73	5.15	26.34	5.90	31.57	5.70	128.34 **	1 < 2,3,4
										2 < 4
										3 < 4
MINI COPE Active coping	2.62	1.68	2.42	1.61	2.26	1.53	2.16	1.54	2.58 *	1 > 4
Planning	2.80	1.65	2.75	1.61	2.42	1.41	2.43	1.77	1.84	
Positive reframing	2.93	1.74	2.61	1.61	1.96	1.73	1.62	1.50	18.90 **	1,2 > 4
Acceptance	3.97	1.46	3.87	1.34	3.50	1.30	3.27	1.71	6.94 **	1,2 > 4
Humour	2.02	1.34	2.16	1.25	1.84	0.88	1.74	1.27	2.44	
Religion	1.08	1.64	1.29	1.81	1.50	2.03	0.92	1.56	1.45	
Use of emotional support	3.37	1.80	3.51	1.56	1.65	1.55	2.37	1.73	17.90 **	1 > 3,4
										2 > 3
Use of instrumental support	2.73	1.76	3.09	1.63	1.73	1.45	2.15	1.63	9.20 **	1 > 3,4
										2 > 3
Self-distraction	3.11	1.56	3.09	1.33	2.38	1.23	2.48	1.46	6.94 **	1 > 4
										2 > 4
Denial	0.91	1.27	1.47	1.62	1.15	1.28	1.74	1.51	8.63 **	1 < 2,4
Venting	2.59	1.54	3.28	1.37	2.76	1.24	2.86	1.57	6.83 **	1 < 2
Substance use	0.64	1.23	0.91	1.40	1.00	1.35	1.43	1.83	9.05 **	1 < 4
										2 < 4
Behavioural disengagement	1.18	1.33	2.27	1.50	2.65	1.29	3.09	1.50	63.30 **	1 < 2,3,4
										2 < 4
Self-blame	1.33	1.36	2.76	1.68	2.46	1.55	3.23	1.80	58.32 **	1 < 2,3,4
										2 < 4 ^1^

1–2; 1–3; 1–4, 2–3, 2–4—significant differences (*p >* 0.05) between groups, * *p* > 0.05, ** *p* < 0.01, ^1^ *p* < 0.1.

## Data Availability

The data presented in this study are available on request from the corresponding author.
